# Application of the BOPPPS teaching model in the teaching of introduction to nursing course

**DOI:** 10.1186/s12912-025-03880-6

**Published:** 2025-09-29

**Authors:** Bei Zhang, Aixu Duan, Jiaqing Lan, Yonglian Ren

**Affiliations:** School of Nursing, Shanxi Datong University, Datong, Shanxi Province China

**Keywords:** BOPPPS teaching model, Autonomous learning ability, Professional identity, Introduction to nursing, Nursing education

## Abstract

**Objective:**

To explore the application effect of BOPPPS (Bridge-in, Objectives, Pretest, Participatory Learning, Post-test and Summary) teaching mode in teaching of introduction to nursing course.

**Methods:**

A convenience sample of 81 undergraduate students from 2 classes of nursing in grade 2023 was assigned as the experimental group, while 69 students from 2 classes of nursing in grade 2022 served as the control group. The control group received traditional lecture-based instruction, whereas the experimental group underwent the BOPPPS teaching model delivered via the Superstar Learning Platform. Comparative analysis evaluated autonomous learning ability and professional identity levels between groups, with further investigation into their intercorrelation.

**Results:**

Students in the experimental group demonstrated significantly higher total scores in autonomous learning abilities, along with elevated subscale scores in learning motivation, planning and implementation, and self-management compared to the control group (*P* < 0.05). Similarly, the experimental group exhibited statistically greater total professional identity scores and improved performance across its cognitive, affective, behavioral, and appropriateness subdimensions (*P* < 0.05). Correlation analysis revealed significant positive associations between autonomous learning abilities (including all subscales) and professional identity dimensions (*P* < 0.01).

**Conclusions:**

The application of BOPPPS teaching mode in the teaching of the introduction nursing course enhances students’ autonomous learning abilities and fosters their professional identity. Furthermore, professional identity and autonomous learning ability exhibit a mutually reinforcing relationship, and the structured framework of the BOPPPS model effectively facilitates their synergistic integration.

## Introduction

The Introduction to Nursing course is a foundational course in nursing education that guides students to understand the basic theories and framework of nursing. It serves as a bridge between the foundational theories of nursing and clinical nursing courses [1]. As the first nursing course for nursing students, it plays a significant role in guiding students’ core values, professional identity, knowledge acquisition, and skill development. While foundational, the Introduction to Nursing course presents significant challenges for novice learners, often leaving them feeling overwhelmed and profoundly confused. This stems not only from the inherent theoretical complexity but also from several key hurdles: navigating a dense influx of unfamiliar terminology without contextual grounding, undergoing a demanding shift in perspective towards professional identity formation involving abstract ethical and value-based concepts, and grappling with the sheer breadth of foundational knowledge spanning diverse domains from history and philosophy to legal aspects. Consequently, students struggle to meaningfully integrate concepts, leading to superficial learning, diminished motivation, and considerable anxiety about their impending professional journey [2, 3]. It is within this context of multifaceted learning difficulties that exploring innovative, structured pedagogical approaches like the BOPPPS model becomes imperative. 

The BOPPPS teaching model was initially proposed in 1978 by Douglas Kerr at the University of British Columbia [4]. Rooted in cognitive theory and constructivism, this framework emphasizes student engagement and feedback, establishing a goal-oriented, student-centered approach focused on effective curricular design [5, 6]. It systematically structures classroom activities into six defined phases: commencing with Bridge-in (contextual transition to activate prior knowledge), followed by explicit Objectives (learning outcome specification); subsequently conducting Pre-assessment (baseline diagnosis of student readiness), then engaging learners through Participatory Learning (active knowledge construction via collaborative methods); immediately followed by Post-assessment (mastery evaluation against predefined objectives), and culminating in Summary (metacognitive consolidation of core concepts)—collectively forming a closed-loop system [7, 8]. Its concise structure and user-friendly implementation have made it particularly valuable for training core faculty in instructional design and frontline teaching practices [9].

The BOPPPS teaching model has been successfully implemented across diverse medical disciplines, spanning basic to clinical medicine, demonstrating significant efficacy in stimulating learning interest, cultivating learner initiative, and enhancing clinical skills [10–12]. In recent years, research on the application of the BOPPPS teaching model within nursing education has demonstrated a significant growth trend. Not only has the volume of such research increased, but the breadth and depth of its exploration have continuously expanded, specifically encompassing: the clinical nursing instruction level, the nursing education level, and the nursing management training level. These studies, through methods such as comparing teaching effectiveness and analyzing learner feedback, consistently demonstrate the positive role of BOPPPS in enhancing the quality, efficiency, and learner satisfaction of nursing-related teaching and training [13–16].

Given the unique challenges of the Introduction to Nursing course—its foundational role in shaping professional identity, the complexity of abstract concepts, and the critical need to effectively engage novice students—the adoption of the structured, student-centered BOPPPS model is imperative. This study aims to bridge the gap between passive learning and active participation inherent in traditional teaching methods, exploring the role of this pedagogical approach in enhancing student motivation, fostering self-directed learning capabilities, and consolidating professional values.

## Materials and methods

### Design

Utilizing a non-concurrent controlled study design, 69 students enrolled in 2022 were assigned to the control group, and 81 students enrolled in 2023 were assigned to the experimental group. The inclusion criteria were as follows: (1) full-time undergraduate nursing students; (2) no prior exposure to the BOPPPS teaching model; (3) provision of informed consent for voluntary participation. Individuals with severe cognitive or emotional disorders were excluded. No significant differences were found in age, gender, only-child status, or admission types between the two groups (all *p* > 0.05), indicating comparable baseline characteristics (see Table 1 for details).

**Table 1:** Comparison of demographic characteristics between the experimental and control groups

Characteristics		Experimental group *(n = 81)*	Control group *(n = 69)*	Z/χ²	*p*-value
Age		18 (19, 20)	18 (19, 20)	-0.243	0.808 ^a^
Gender	Male	18 (22.2%)	14 (20.3%)	0.083	0.773^b^
	Female	63 (77.8%)	55 (79.7%)		
Only-child status	Yes	9 (11.1%)	12 (17.4%)	1.221	0.269 ^b^
	No	72 (88.9%)	57 (82.6%)		
Admission Type	First-Choice	52 (64.2%)	33 (47.8%)	5.343	0.069^b^
	Non-First Choice	23 (28.4%)	24 (34.8%)		
	Program Transfer	6 (7.4%)	12(17.4%)		

Note: a: The two groups were compared using the Mann-Whitney U test. b: The two groups were compared using the Chi-square test

### Educational intervention

#### Teaching method of control group

The control group received traditional teaching methods. The textbook used was *Introduction to Nursing* (5th Edition) published by People’s Medical Publishing House. The teaching was conducted through a blended online-offline format, with online components administered via the Superstar Learning Platform primarily for attendance checks ten minutes before class and post-class homework distribution for knowledge consolidation. In class, instruction remained predominantly lecture-based, with occasional sporadic, unstructured question-and-answer exchange activities initiated by students. The pedagogical approach excluded all preparatory learning tasks, structured interactive activities and real-time assessment mechanisms, positioning technology solely as an auxiliary instrument.

#### Teaching method of experimental group

The teaching team consisted of instructors with over five years of experience teaching introduction to nursing course, all of whom received systematic training in BOPPPS theory and teaching skills. The experimental group used the same textbook and was taught by the same teaching team as the control group. The theoretical course hours and content distribution were identical.

The BOPPPS model was applied to the two-hour weekly teaching content. The implementation of each stage was conducted either online via the Superstar Learning Platform or offline in the classroom, depending on the specific teaching content. These standardized functions—such as automated attendance tracking, notification alerts, assignment reminders, and grading—were systematically implemented via the Superstar Learning Platform to ensure intervention fidelity by maintaining student attendance and sustaining active classroom engagement. The Bridge-in stage often used cases, current events, social hot topics, or data to introduce the topic, which could be released 2–3 days before class as a “task point” on the learning platform or presented in the first 2–3 min of class using PPT with images and text. Learning Objectives are mainly given in the form of PPT presentation combined with teacher’s explanation in class, and for memorization-based objectives, they could also be released after the Bridge-in activities. Pre-assessment was conducted through quizzes, questions, polls, or questionnaires, either in class or via the learning platform before class. Participatory Learning focused on engaging students through various teaching activities such as Q&A, peer sharing, group work, and role-playing. Post-assessment was designed based on the learning objectives, with quizzes for memorization-based objectives and case analysis for application-based objectives. The Summary stage involved the teacher reviewing key content, establishing logical relationships between knowledge points, and extending the application of the learned content, aiming to help students consolidate learning outcomes. Table 2 illustrates the implementation process of the BOPPPS model in the Introduction to Nursing course, using the chapter “End-of-Life Care” as an example. (see Table 2 for details).

**Table 2:** Implementation process of BOPPPS in the teaching of introduction to nursing course (Example of a Chapter)

BOPPPS	Timelines	Teacher activities	Student activities	Purpose	Teaching equipment
Bridge-in	2 to 3 days prior to the scheduled instructional session	Post learning tasks on the Superstar Learning Platform, require students to watch excerpts from the documentary Life Out of Control to guide them in understanding and experiencing the hardships and suffering experienced by terminally ill patients.	Video viewing and critical Thinking	Stimulate students’ interest in the current unit content and facilitate their focus on the upcoming instructional materials to be presented by the teacher.	Superstar Learning Platform
Objectives	In class (5 min)	Teach + PPT	Listen and think	Clearly define the learning objectives in terms of cognition, skills and emotions for this unit, so as to enable students to better understand the interrelationships among activities.	Microsoft PowerPoint
Pre-assessment	In class (10 min)	Center on content regarding hospice care development from the related policies; Listen and repeat students’ understanding of hospice care or palliative care.	Share one’s understanding, attitude, viewpoints regarding palliative care.	Assess students’ prior knowledge and competencies, thereby informing instructional strategies and enabling timely adjustments to content depth and pacing of instruction.	Microsoft PowerPoint
Participatory Learning	In class (70 min)	Combines didactic lectures, Socratic questioning, and systematic synthesis to investigate social-psychological theories and their practical applications in death studies and hospice care. *Curriculum Ideology and Politics Integration*: employ case-based pedagogy, aiming to facilitate students’ critical reflection on the existential value of life.	Role-Play and Group Collaboration; Think-Pair-Share (TPS); Peer Learning	Enable students to actively experience, explore and learn effectively in specific scenarios.	Microsoft PowerPoint
Post-assessment	In class (10 min)	In-Class Quiz (Case Analysis) + Results Feedback (Distribution and Statistics via Superstar Learning Platform)	Answer the question	Evaluate students’ achievement of intended learning outcomes and ascertain the attainment of predefined educational objectives through a formative evaluation framework.	Superstar Learning Platform
Summary	In class (5 min)	Review of Key Content and Assign Post-Class Study. *Case Example & Curriculum Ideology and Politics Integration: Watch or present videos/data related to the development of hospice care services, guiding students to cultivate professional ethics, with the aim of fostering reverence and respect for life and inspiring them to become outstanding nursing professionals.	Summary + Listening and Note-Taking	Enable students to visualize their academic progress and enhance their mastery of the learning content through systematic summarization and synthesis processes.	Microsoft PowerPoint

### Effectiveness assessment

At the end of the course, quantitative data were collected using the Autonomous Learning Ability Scale for Nursing Students and Professional Identity Questionnaire for College Students. Additionally, correlation analysis between autonomous learning ability and professional identity was explored.

### Measurement of students’ autonomous learning ability

The Autonomous Learning Ability Scale for Nursing Students [17] was used for evaluation. The scale includes 4 dimensions and 20 items, scored on a Likert 5-point scale from “strongly disagree” to “strongly agree,” with a total score ranging from 10 to 100. A score of 60 is considered passing, with higher scores indicating stronger autonomous learning ability. In this study, the scale demonstrated Cronbach’s α coefficients of 0.718–0.773 for individual dimensions and 0.931 for the full scale.

### Measurement of students’ students' professional Identity

The Professional Identity Questionnaire for College Students [18] was used for measurement. The questionnaire includes 4 dimensions (cognitive, affective, behavioral, and appropriateness) and 23 items, scored on a Likert 5-point scale from “completely disagree” to “completely agree.” Higher scores indicate higher professional identity. In this study, the scale demonstrated Cronbach’s α coefficients of 0.760–0.885 for individual dimensions and 0.943 for the full scale.

### Data analysis

Statistical analysis was performed using SPSS software (version 22.0). The normality of continuous variables was assessed with the Shapiro-Wilk test. Depending on the data distribution, results are presented as median with interquartile range (IQR). Group comparisons were conducted using the Mann-Whitney U test, while categorical variables were analyzed with the chi-square test. All tests were two-sided, with significance set at *p* < 0.05.

## Results

### Result analysis of students’ autonomous learning ability and its dimension scores

The results of pairwise comparisons demonstrated that the total score of autonomous learning ability and the scores of learning motivation dimension, planning and implementation dimension, and self-management dimension of students in the experimental group were higher than those of the control group, and the difference was statistically significant (*p* < 0.05). The difference in interpersonal communication ability scores is not statistically significant (*p* > 0.05) (see Table 3 for details).

**Table 3:** Comparison of autonomous learning ability scores between the two groups

Autonomous learning ability	Experimental group *(n = 81)*	Control group *(n = 69)*	Z	*p*-value
Learning motivation	23 (18, 24)	21 (18, 24)	-2.108	0.035
Planning and implementation	23 (18, 24)	20 (18, 24)	-2.366	0.018
Self-management	16 (13, 17)	14 (12, 16)	-3.393	0.001
Interpersonal communication	16 (12, 17)	15 (12, 16)	-1.904	0.057
Total score	77 (61.5, 81)	70 (61, 77.5)	-2.854	0.004

Note: The two groups were compared using the Mann-Whitney U test

### Result analysis of students’ professional identity and its dimension scores

Pairwise comparisons revealed that the total score of professional identity scale and the scores of their cognitive dimension, affective dimension, behavioral dimension, and appropriateness dimension of students in the experimental group were higher than those of the control group, and the difference was statistically significant (*p* < 0.05) (see Table 4 for details).

**Table 4:** Comparison of professional identity scores between the two groups of students

Professional identity	Experimental group *(n = 81)*	Control group *(n = 69)*	Z	*p*-value
Cognition	20(18.5, 21)	19(17, 20)	-2.610	0.009
Emotionality	32(25.5, 33)	30(25, 32)	-2.434	0.015
Behavioral	24(21, 25)	23(18, 24)	-2.631	0.009
Fitness	16(13,17)	14(12, 16)	-3.037	0.002
Total score	91 (80, 94)	84(72, 91)	-3.288	0.001

Note: The two groups were compared using the Mann-Whitney U test

### Results of the correlation analysis between autonomous learning ability and professional identity

Correlation analysis showed that students’ self-directed learning ability and all its dimensions were significantly and positively correlated with professional identity and its dimensions (*p* < 0.01) (see Table 5 for details).

**Table 5:** Correlation analysis between autonomous learning ability and professional identity

	Learning motivation	Planning and implementation	Self-management	Interpersonal communication	Autonomous Learning Ability	Cognition	Emotionality	Behavioral	Fitness	Professional Identity
Learning motivation	1.000									
Planning and implementation	0.861^**^	1.000								
Self-management	0.739^**^	0.844^**^	1.000							
Interpersonal communication	0.692^**^	0.789^**^	0.811^**^	1.000						
Autonomous learning ability	0.907^**^	0.951^**^	0.905^**^	0.876^**^	1.000					
Cognition	0.541^**^	0.570^**^	0.475^**^	0.514^**^	0.558^**^	1.000				
Emotionality	0.573^**^	0.555^**^	0.461^**^	0.511^**^	0.551^**^	0.669^**^	1.000			
Behavioral	0.652^**^	0.633^**^	0.554^**^	0.551^**^	0.638^**^	0.658^**^	0.790^**^	1.000		
Fitness	0.611^**^	0.677^**^	0.550^**^	0.568^**^	0.640^**^	0.643^**^	0.817^**^	0.808^**^	1.000	
Professional Identity	0.657^**^	0.667^**^	0.572^**^	0.600^**^	0.664^**^	0.782^**^	0.936^**^	0.898^**^	0.916^**^	1.000

Note: ‘**’: *p* < 0.01

## Discussion

### Improvements in students’ autonomous learning ability

Autonomous learning ability emphasizes the initiative of the learner and is a core competency for improving learning strategies, enhancing learning outcomes, and fostering lifelong learning among undergraduate nursing students [19]. The Introduction to Nursing course is typically taken by freshmen who are still transitioning from high school to university. Many students lack initiative and autonomous thinking skills, and the theoretical nature of the course further affects their learning experience and performance. Therefore, improving students’ autonomous learning ability is one of the key objectives of this course. The study shows that the overall scores of autonomous learning ability of students in the experimental group are higher than those of the control group, in which the students’ motivation, plan implementation, and self-management ability are significantly improved, and the difference in interpersonal communication ability scores is not statistically significant. The BOPPPS teaching model is fundamentally rooted in fostering student independence [20], characterized by its emphasis on students’ self-directed acquisition of learning-related information through various modalities, including pre-class reading, in-class group discussions, and post-class reinforcement activities [8]. This framework effectively enhances learning initiative, clarifies learning objectives, and promotes task-driven engagement in pedagogical practices. More importantly, the BOPPPS model intrinsically embodies the principles of Self-Regulated Learning (SRL): the Bridge-in phase translates course objectives into personal goals (Planning phase); the Pre-test/Participatory activity facilitates real-time strategy adjustments (Execution phase); and the Post-test phase reinforces metacognitive awareness (Reflection phase) [21]. Through effective organization and guidance, teachers facilitate students’ better application of theoretical knowledge to real-world situations by incorporating real cases and simulated practices [7], so that students can acquire knowledge, master skills, feel professional culture. And in the continuous practice, teaching activities as external learning motives continuously stimulate students’ internal learning motives, so that students’ learning motives are stimulated and maintained, laying the foundation for further programme implementation and self-management. In this study, no significant difference in the interpersonal communication skills of the two groups of research subjects was observed, which may be related to the fact that there were not enough group co-operation activities designed in the teaching activities or that the activities were not in-depth enough due to the time of the lectures, which is similar to the results of the study conducted by Tan YB [22]. SRL theory further explains this phenomenon: inadequate collaborative depth restricts the mechanism of observational social learning, which is a crucial element for acquiring communication skills.

### Improvements in students’ professional identity

Professional identity refers to nursing students’ recognition of the profession’s intrinsic value, accepting the profession from the bottom of their hearts, and making positive perceptions and positive evaluations of all aspects of the profession [23]. As the first professional course that nursing students study, Introduction to Nursing course is strategically positioned to cultivate professional identity among students through systematic exposure to disciplinary paradigms and value internalization processes. In this study, the BOPPPS model naturally matches Social Identity Theory (Tajfel & Turner, 1979), which strengthened this cultivation process [24]. The results indicate that students in the experimental group have higher professional cognition, emotionality, behavioral and fitness than those in the control group. This enhancement stems from the design of the BOPPPS teaching model. The Bridge-in component, by establishing nursing roles, guides students through social categorization, prompting them to self-categorize within the professional nursing group. The Participatory Learning component, utilizing methods such as group collaboration, case analysis, and role-playing, effectively fostered students’ sense of identification with the professional group. Consequently, the BOPPPS not only helps students better understand professional knowledge but also enhances their perception, understanding, and adaptation to the profession, leading to emotional and behavioral recognition, which is consistent with the results of Wang Rui et al. in the application of nursing management course [25].

### Professional identity and autonomous learning ability mutually reinforce each other, and the BOPPPS teaching mode helps the two complement each other better

Correlation analysis shows that nursing students’ autonomous learning ability and its dimensions are significantly positively correlated with professional identity and its dimensions. This suggests that appropriate teaching strategies or models can enhance students’ autonomous learning ability and stimulate their professional identity. At the same time, the design of participatory activities in the BOPPPS model helps students better understand and appreciate the nursing profession, fostering emotional and cognitive recognition of the profession. This, in turn, motivates students to align their learning goals with the profession, thereby strengthening their autonomous learning behavior and ability. In other words, professional identity positively predicts learning motivation, which influences autonomous learning ability [26]. Additionally, increased learning engagement, such as planning and implementation, self-management, and interpersonal communication, also plays a role [27, 28]. *Introduction to Nursing* course is the first professional course for nursing students to learn, and it is also a basic course that guides nursing students to comprehensively understand the connotation and characteristics of the nursing profession, the core value system of the profession, the knowledge of the main disciplines and the curriculum system, and the relationship between the profession and the social and economic development of the society, which has the characteristics of strong theoretical and abstract content. This study implemented the BOPPPS teaching mode, theoretically anchored in Bloom’s Classification of Educational Objectives, through a pedagogical design encompassing pre-class, in-class, and post-class modules. We guide students to start from life experiences, social hotspots, and data, enabling them to concentrate on learning content. By setting observable and measurable learning objectives, we help students target their efforts and grasp key learning priorities. Through conducting the pre-test to understand the students’ learning situation, the teaching content was adjusted in terms of its depth and pace in a timely manner, enabling most students to master the knowledge relatively well. Participatory learning engages students through a variety of classroom activities to allow students to ‘move’, so that students closely follow the teacher’s teaching rhythm. The post-test and summary help students integrate knowledge points and master the course content and key points. The whole teaching mode can make the teaching content and students’ existing knowledge fit as much as possible, which makes abstract and complex theoretical knowledge more concrete and vivid, while helping students focus on key learning points from the start, thereby improving teaching effectiveness.

### Limitations

This study is subject to three primary limitations. Firstly, the single-institution design, while enhancing internal consistency through standardized resources, limits generalizability to diverse educational contexts Secondly, assessment confined to one semester (16 weeks) precludes evaluation of longitudinal outcomes. Thirdly, potential residual confounding persists from unmeasured factors including participants’ prior medical exposure and learning style variations. Future research should employ multicenter randomized controlled trials with larger cohorts, actively measure key confounders via validated tools, and extend follow-up periods to evaluate the intervention’s sustained efficacy.

## Conclusions

The BOPPPS teaching model is a student-centered and effective teaching approach that significantly enhances students’ autonomous learning ability and professional identity. Moreover, the two aspects mutually reinforce each other: improved autonomous learning ability leads to deeper engagement and understanding of the profession, while enhanced professional identity motivates students to actively learn and explore, further boosting their autonomous learning ability.

## Data Availability

The datasets used during the current study are available from the corresponding author on reasonable request.
